# Supplementary material for: Staphylococcus aureus produces pain through pore-forming toxins and neuronal TRPV1 that is silenced by QX-314
Source: Nat Commun. 2018 Jan 2;9:37. doi: 10.1038/s41467-017-02448-6 (PMC5750211; doi:10.1038/s41467-017-02448-6)
Supplement: Supplementary file 1 — Supplementary Information [file 41467_2017_2448_MOESM1_ESM.pdf]

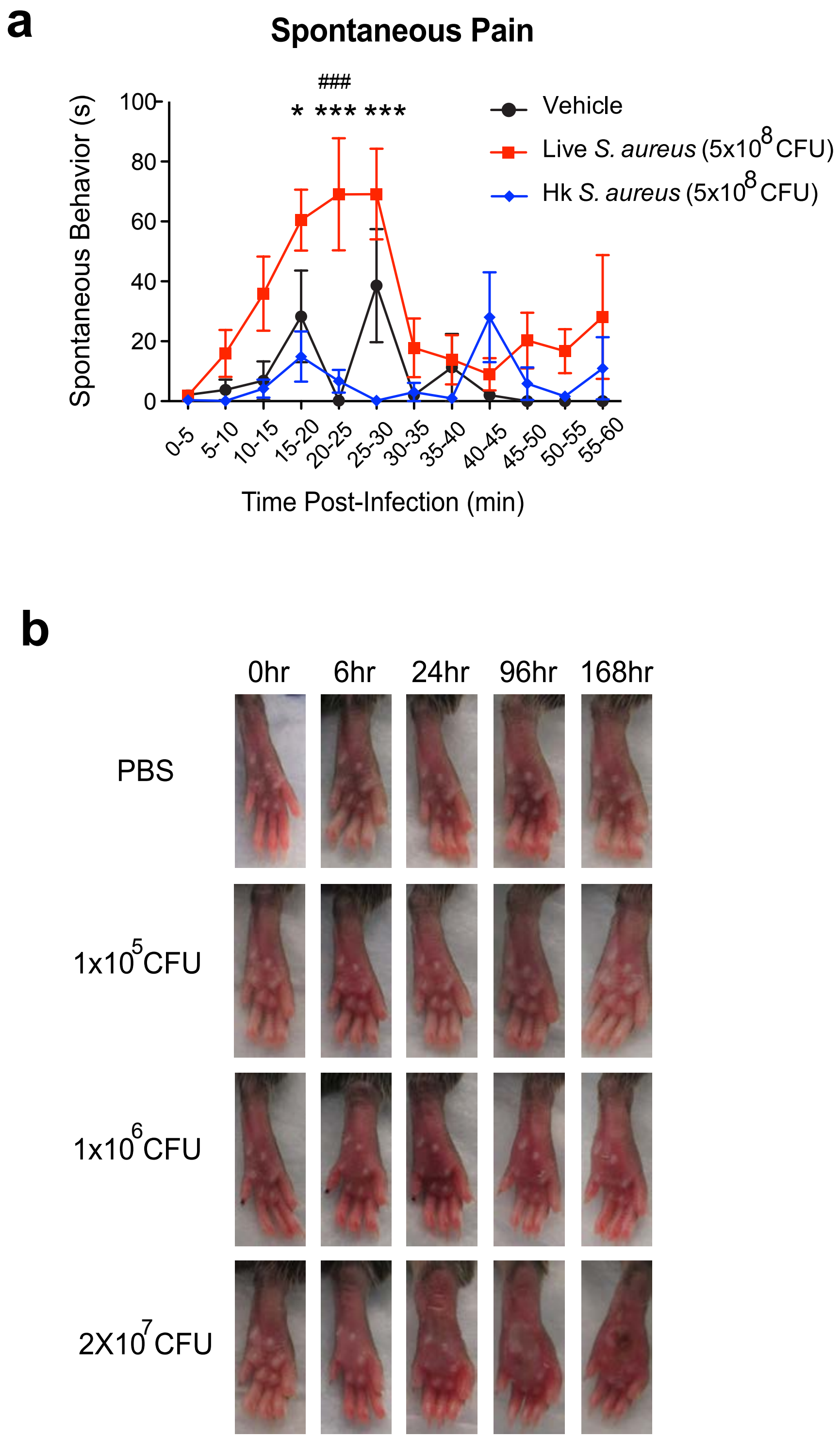

Supplemental Figure 1. *S. aureus* induced spontaneous pain time course and representative pictures of *S. aureus* infection. a. Spontaneous pain time course in 5-minute intervals produced by *S. aureus* infection (USA300, $5x10^8$ CFU, *n*=10 mice/group) vs. Heat-killed (HK) *S. aureus* ($5x10^8$ CFU), *n*=8 mice/group and vehicle (PBS), *n*=9 mice/group. *N*=3 replicates. Statistical comparison by two-way ANOVA with Tukey's post-test; *Live *S. aureus* vs PBS; # Live vs HK *S. aureus.* Error bars, mean +/- s.e.m. *** $p<0.001$, ** $p<0.01$, * $p<0.05$. **b.** Representative pictures of time course of infection over 168 hrs by different doses of *S. aureus* (USA300). Of note, the $2x10^7$ CFU *S. aureus* dose induces tissue necrosis but not lower doses.

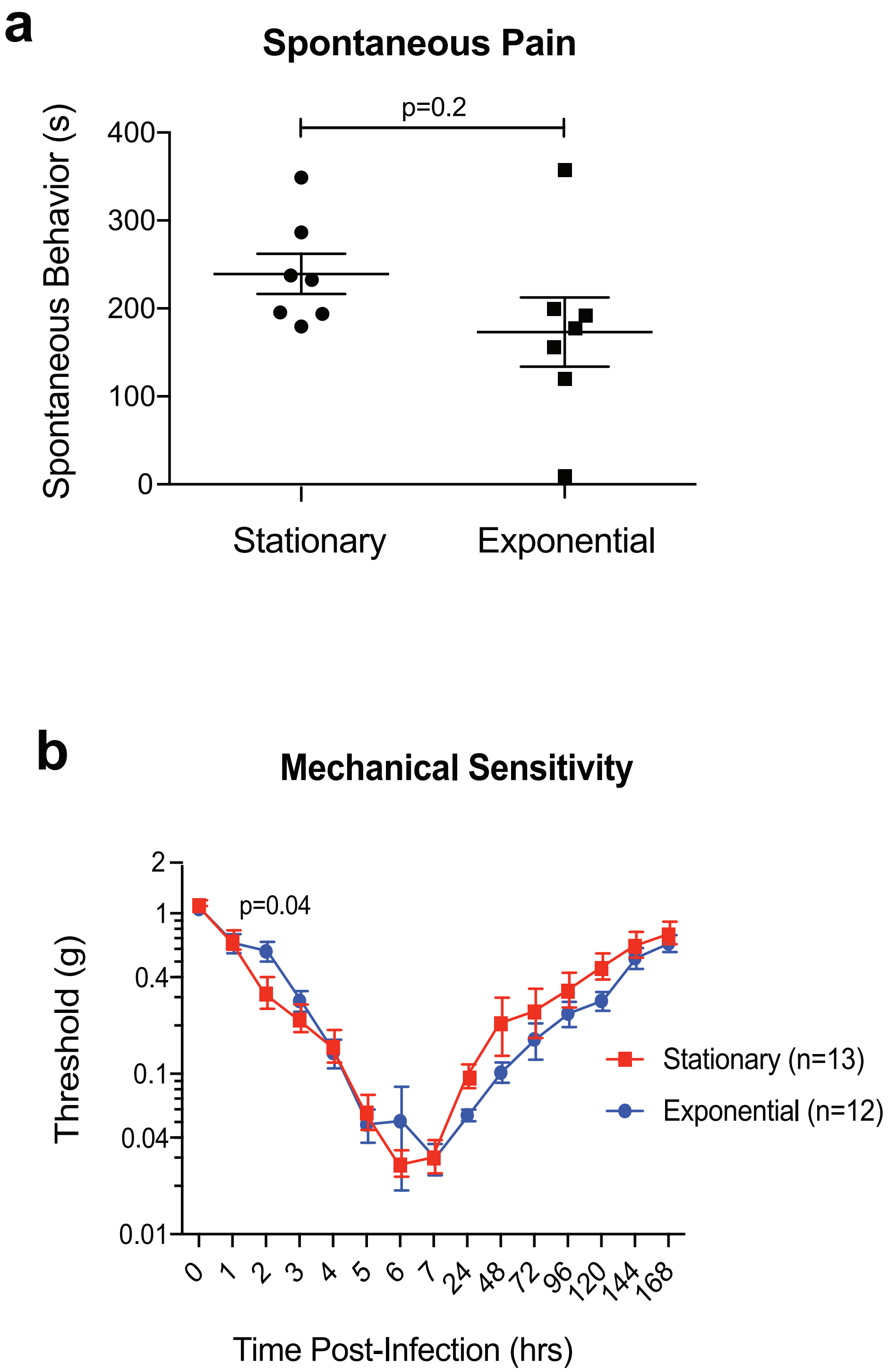

Supplemental Figure 2. Stationary phase and exponential phase *S. aureus* both induce spontaneous pain and mechanical hyperalgesia. a. Spontaneous pain quantified over 60 minutes after infection with stationary or exponential phase *S. aureus* (USA300, $5x10^8$ CFU). There is no difference in spontaneous pain behaviors produced by *S. aureus* at different phases of growth. *n*=7 mice/group. *N*= 3 replicates. *p* values, unpaired t-test. **b.** Mechanical sensitivity measured by von Frey hairs after infection with exponential or stationary phase *S. aureus* (USA300, $1x10^6$ CFU). *n*= 12-13 mice/group. N= 1 replicate. Two-way ANOVA, Sidak's post-test. Error bars throughout figure, mean +/- s.e.m.

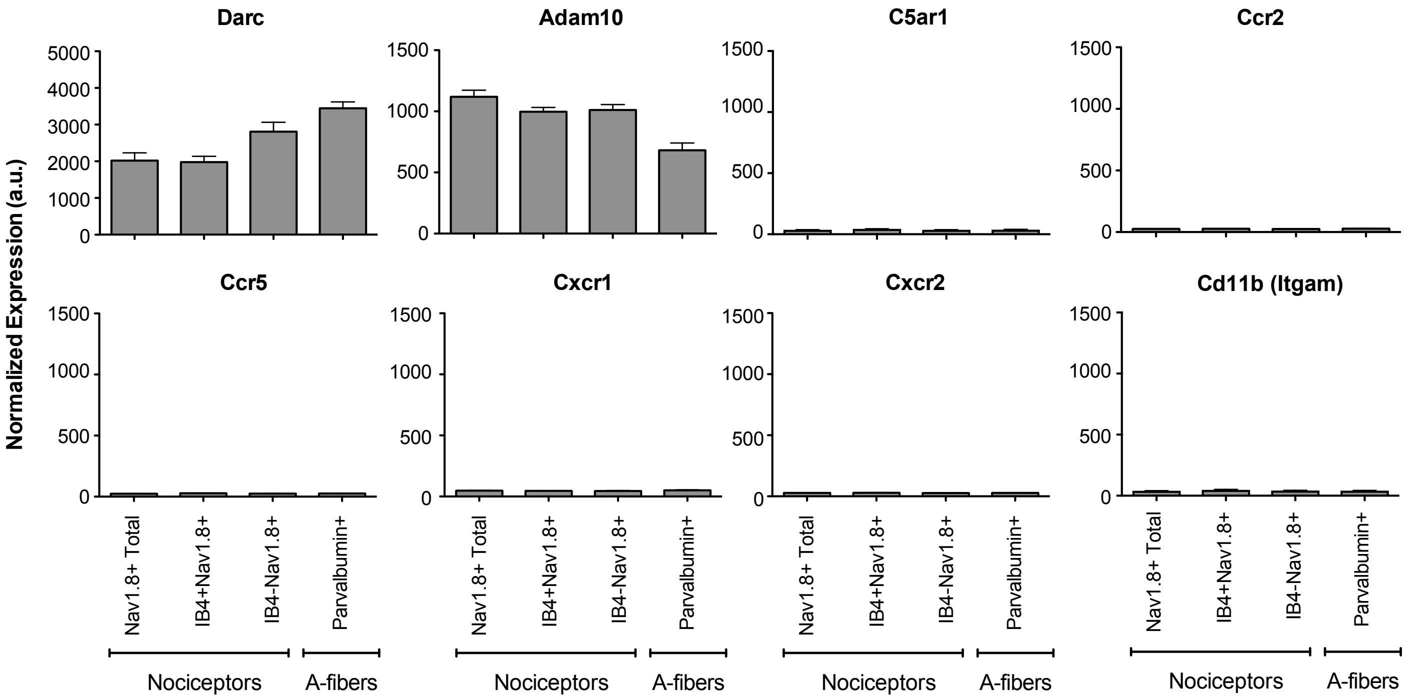

Supplemental Figure 3. Darc and ADAM10, cellular receptors for pore-forming toxins are expressed by nociceptor neurons. FACS purified DRG neuron subsets, including total Nav1.8-cre/TdTomato+ nociceptors, the IB4+Nav1.8-cre/TdTomato+ nociceptor subset, the IB4-Nav1.8-cre/TdTomato subset, and Parv-cre/TdTomato+ proprioceptors, were analyzed for gene expression by microarray analysis (For details, see Chiu et al, 2014). RNA-normalized expression levels of known receptors for *S. aureus* pore-forming toxins are plotted (Error, mean+/-s.e.m.) In general, normalized expression values below 100 are considered absent. *Darc*, one of the receptors for HlgAB and LukED, is expressed by DRG neurons, and *Adam10*, the receptor for Hla, is also expressed in nociceptor neurons. By contrast, *C5ar1, Ccr2, Ccr5, Cxcr1, Cxcr2*, and Cd11b (*Itgam* is the gene name)*,* other leukocidin receptors, are not expressed by neurons.

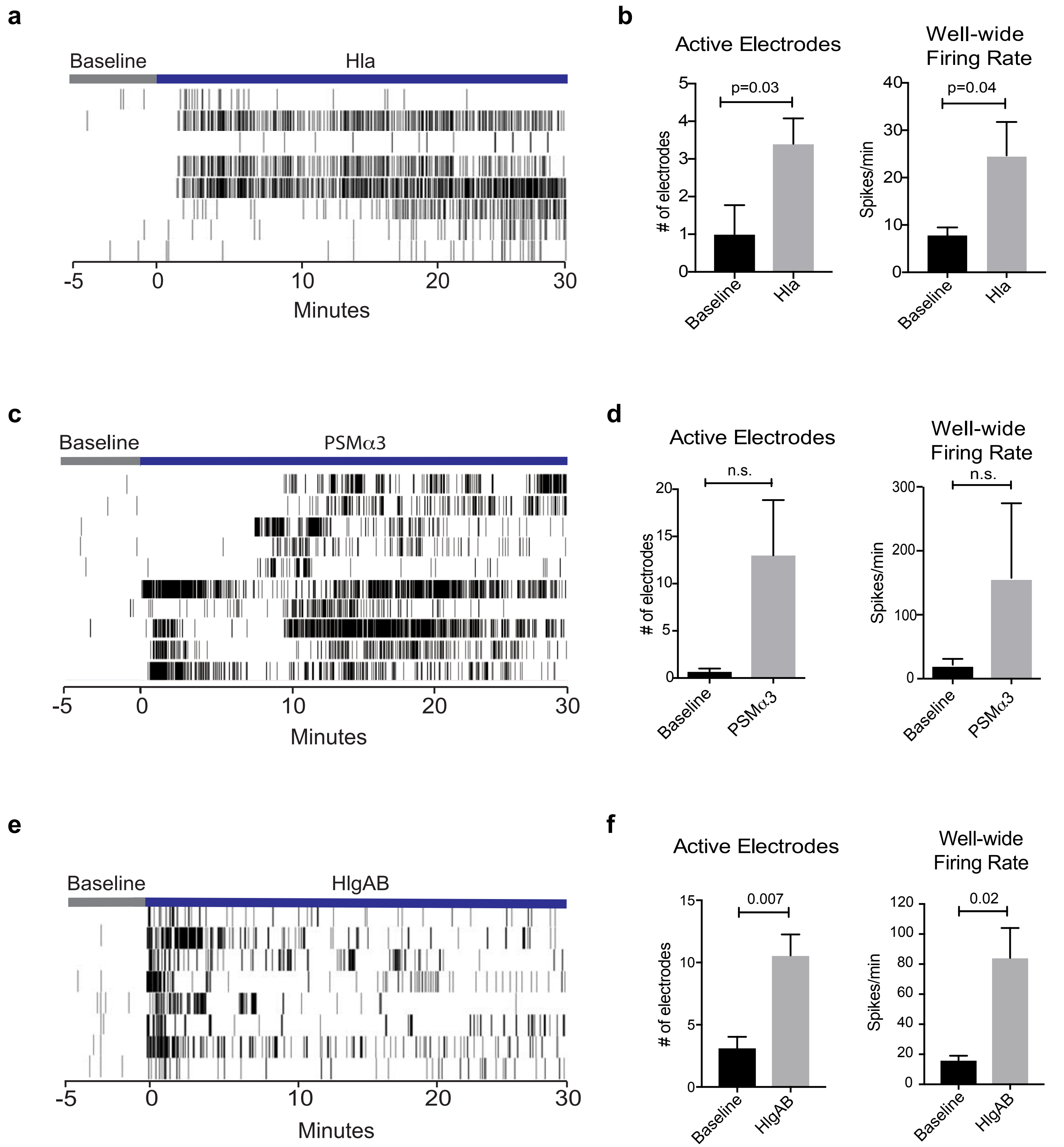

Supplemental Figure 4. Raster plots and quantification of pore-forming toxin induced neuronal firing. **a, c, e** Raster plots showing spike activity (vertical lines are individual action potentials) of representative active electrodes from multi-electrode array (MEA) plates treated with pore-forming toxins (PFT), including Hla, PSMα3, or HlgAB. **b, d, f**. Quantification of DRG neuronal firing before and after indicated PFT addition by measuring the number of active electrodes/plate (>5 spikes/minute) or well-wide firing rate. *p*-values by paired t-test; Hla, 1 μM (30 μg/mL) or, *n*=17 electrodes over 5 plates; PSMα3, 10 μM (270 μg/mL), *n*=41 electrodes over 3 plates; HlgAB, 0.1 μM (3 μg/mL), *n*= 74 electrodes over 7 plates. All experiments done in triplicate. Error bars, mean +/- s.e.m.

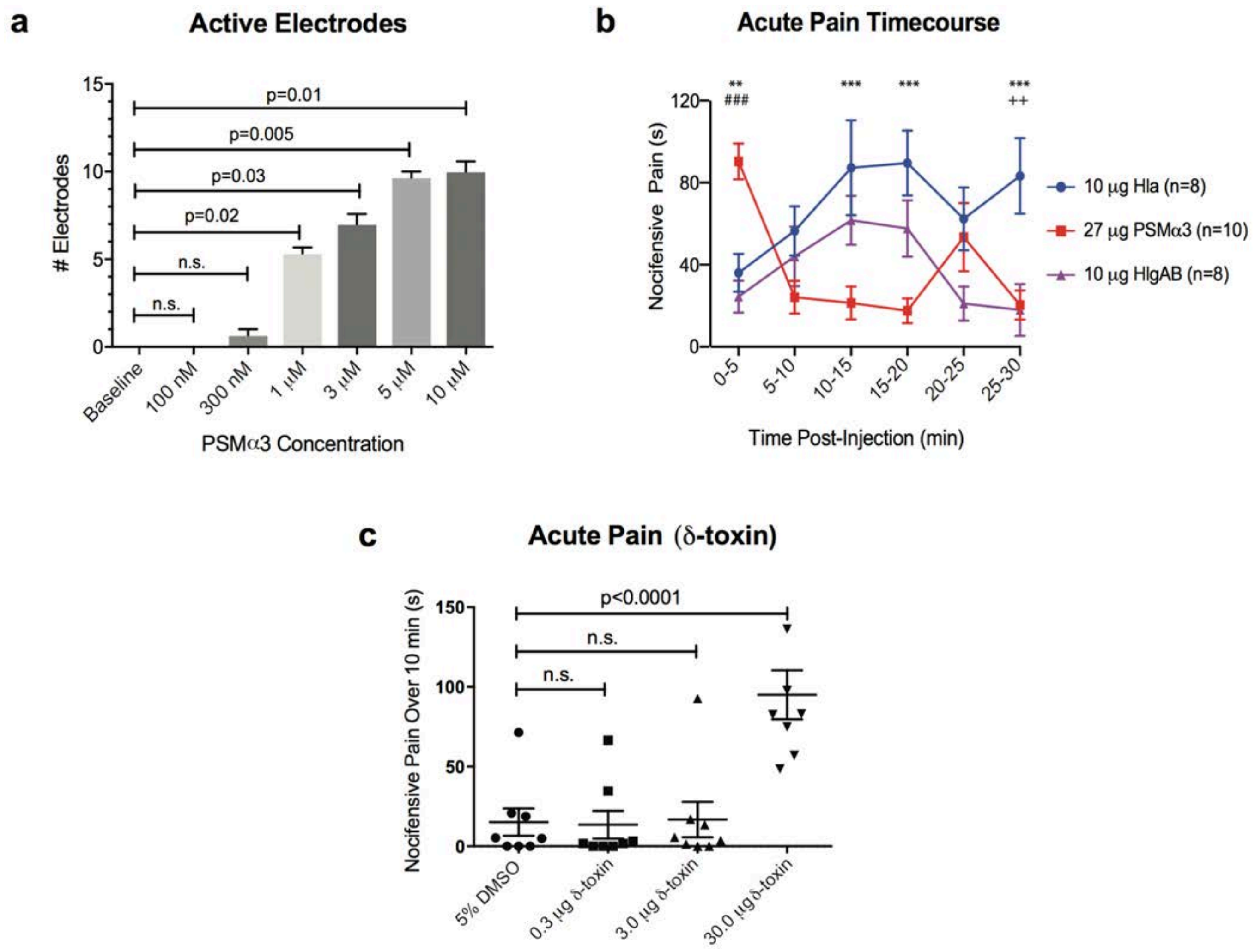

Supplemental Figure 5. Phenol soluble modulins cause dose-dependent neuronal firing *in vitro* and spontaneous pain *in vivo.* a. Increasing doses of PSMα3 were added to DRG neurons cultured on MEA plates. An active electrode was defined as 5 spikes/minute or more in any one minute interval (10 minutes total at each dose), *n*=3 plates. *N*= 2 replicates. P-values, one-way ANOVA, RM, Tukey's post-test. **b.** Time course of spontaneous pain quantified in five-minute intervals after injection with Hla (10 μg, 17 μM), PSMα3 (27 μg, 50 μM), or HlgAB (10 μg, 17 μM). PSMα3 induces immediate spontaneous pain, while HlgAB and Hla spontaneous pain peak around 10 minutes. *n*= 8-10 mice/group. *N*= 2-3 replicates per toxin. *Hla vs PSMα3 comparison; # HlgAB vs. PSMα3; + Hla vs HlgAB. ****$p<0.0001$, ***$p<0.001$, **$p<0.01$, *$p<0.05$. **c.** Increasing doses of δ-toxin, a member of the PSMs, was injected into mice and spontaneous pain quantified over 10 minutes. (5 μM, 50 μM, 150 μM) *n*=8 mice/group. *N*= 3 replicates. *p*-values by one-way ANOVA with Tukey's post-tests. Error bars throughout figure, mean +/- s.e.m.

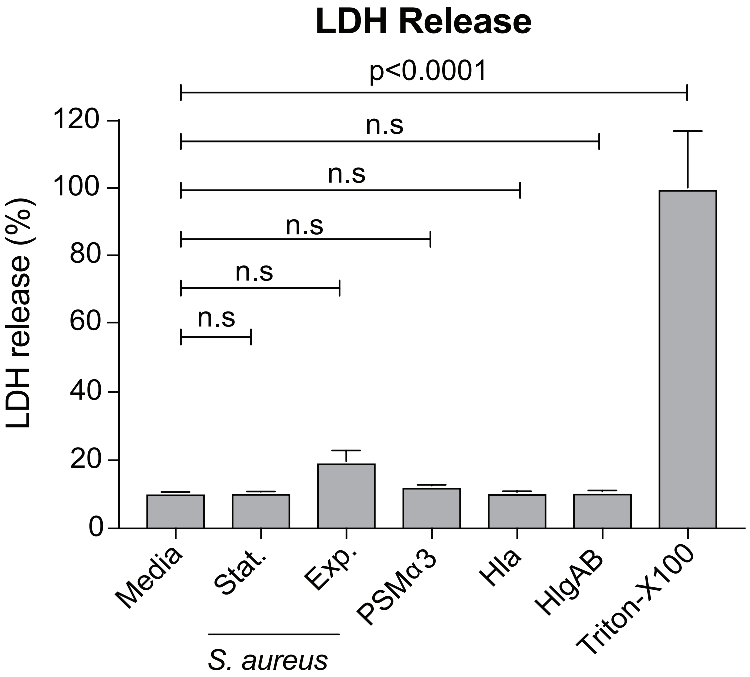

Supplemental Figure 6. Treatment with pore-forming toxins or live *S. aureus* does not induce significant neuronal lysis at early time points. LDH release from DRG neurons (5000 cells/well) was measured after 15 minutes of incubation with indicated pore-forming toxins or with stationary (Stat.) or exponential phase (Exp.) *S. aureus* (Concentrations used: 10 μM (270 μg/mL) PSMα3; 1 μM (30 μg/mL) Hla; 0.1 μM (3 μg/mL) HlgAB; $1x10^9$ CFU/mL *S. aureus*). Triton-X 100 treated neurons represent 100% lysis. *n*= 3 wells per condition. *N*=1 replicate. *p*-values by one-way ANOVA with Tukey's post-tests. Error bars, mean +/- s.e.m.

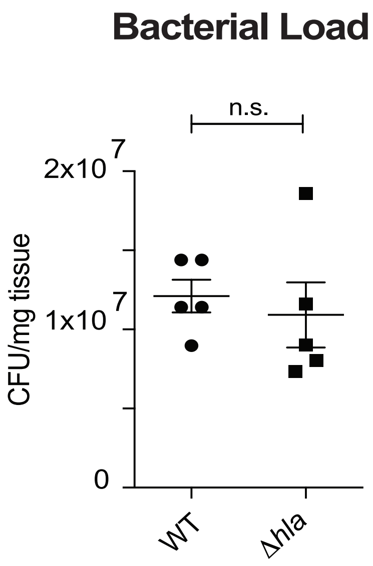

Supplemental Figure 7. The attenuation of spontaneous pain in the absence of Hla (Δ*hla*) was not due to decreased bacterial expansion. Paw tissue was collected, homogenized, and plated after spontaneous pain analysis at the 60-minute time point post-infection by WT or Δ*hla S. aureus* (USA300, $5x10^8$ CFU). Bacterial load recovery was counted and normalized as CFU per mg tissue. Bacterial load did not differ significantly between WT and *Δhla S. aureus* by unpaired t-test. *n*=5 mice/group. *N*=1 replicate. Error bars, mean +/- s.e.m.

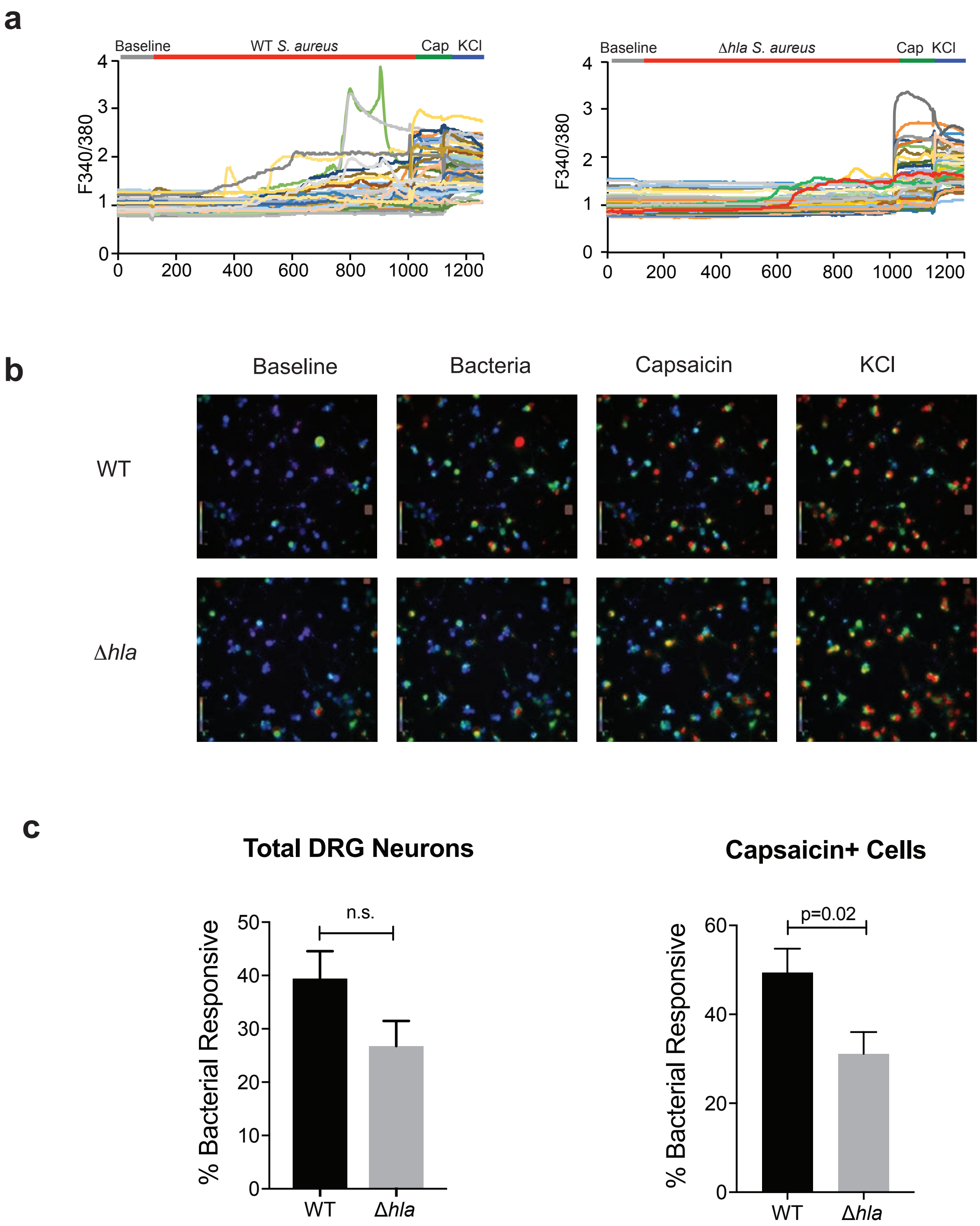

Supplemental Figure 8. *S. aureus* deficient in α-hemolysin (Δ*hla*) shows decreased activation of capsaicin sensitivity nociceptor neurons. a. Representative Fura-2 calcium imaging traces of DRG sensory neurons exposed to live WT or Δ*hla S. aureus* (USA300, $1x10^9$ CFU/mL), followed by capsaicin (1μM), and KCl (40mM). **b.** Representative calcium imaging fields of neurons described in **a**. **c.** Percentage of total neurons (KCl responsive, right) or capsaicin+ neurons (left) responsive to WT or to Δ*hla S. aureus*. Percentage of capsaicin+ DRG neurons responsive to Δ*hla S. aureus* is decreased compared to WT *S. aureus*. *p*-values by unpaired t-test. *n*=9 fields over 3 separate experiments. Error bars, mean +/- s.e.m.

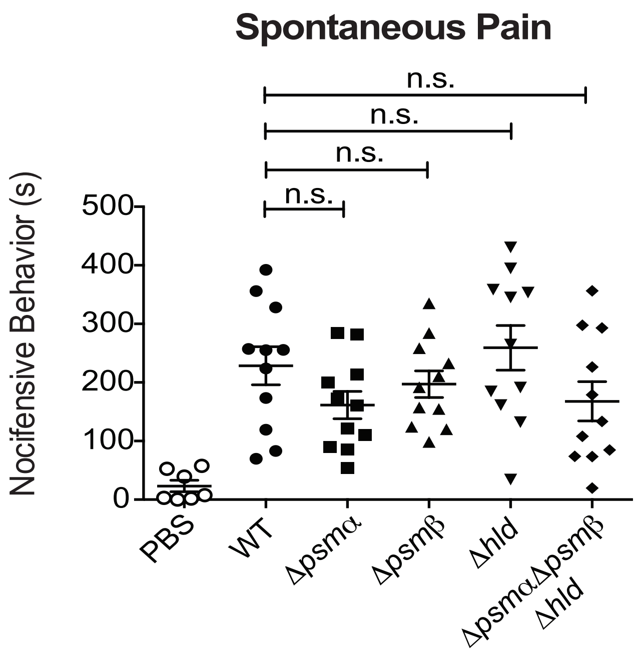

Supplemental Figure 9. Spontaneous pain during *S. aureus* infection is not due to phenol soluble modulins. Spontaneous pain was quantified over 60 minutes after infection with WT or specific isogenic mutant strains lacking PSMα (Δ*psmα*), PSMβ (Δ*psmβ*), delta-toxin (Δ*hld*), or all PSMs (Δ*psmα*Δ*psmβ*Δ*hld*). There was no statistical difference between each mutant strain and WT *S. aureus*. PBS, *n*= 7mice/group; *S. aureus* strains, *n*=11 mice/group. *N*= 4 replicates. *p*-values by one-way ANOVA with Tukey's post-tests. Error bars, mean ± s.e.m.

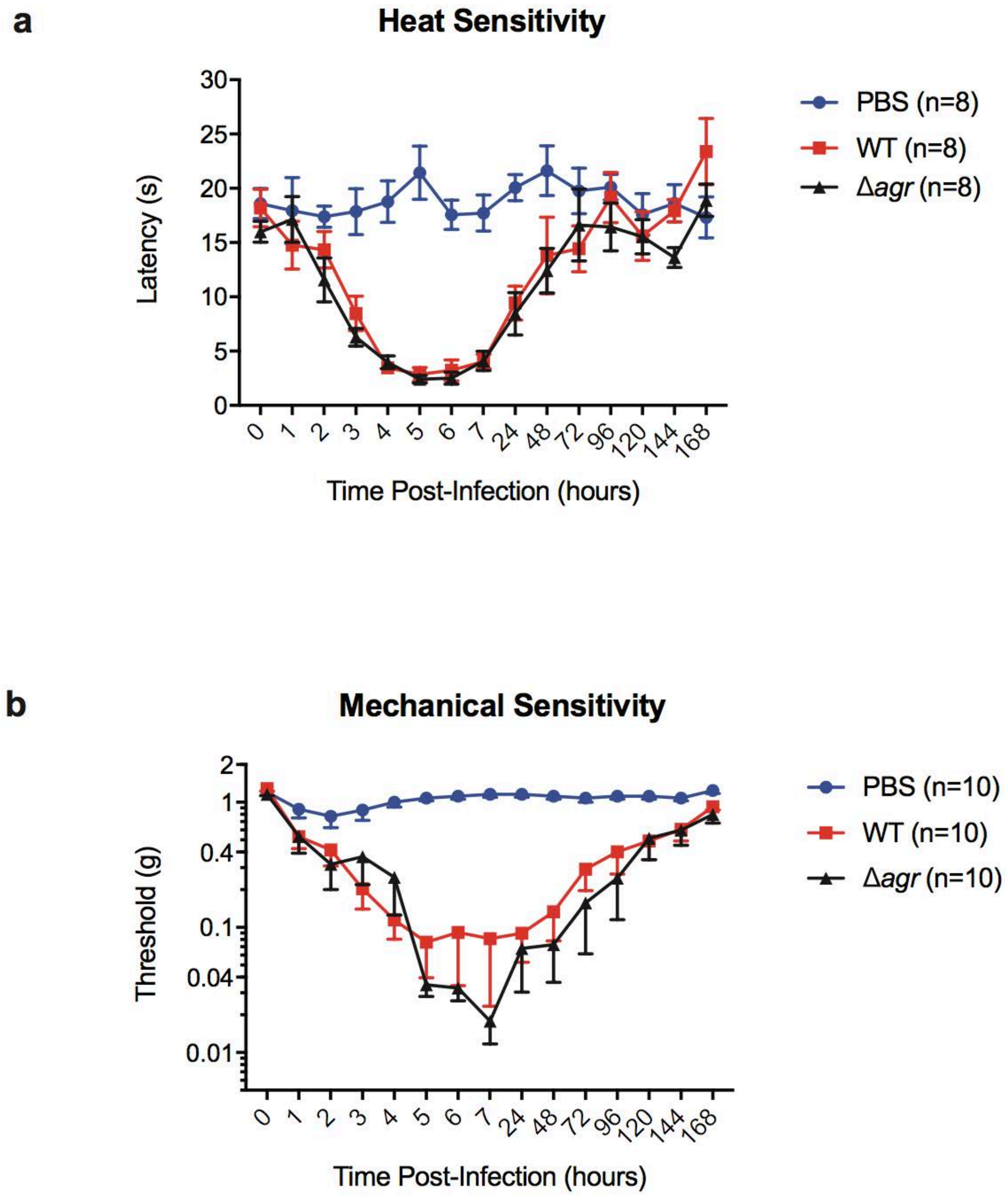

Supplemental Figure 10. The *S. aureus agr* quorum sensing locus contribute to heat or mechanical sensitivity during infection. a, b. Heat and mechanical sensitivity were assayed after infection with WT or Δ*agr S. aureus* (USA300, 1x10^6 CFU). *S. aureus* induced mechanical hyperalgesia, measured by von Frey hairs, and thermal hyperalgesia, measured by the Hargreaves' test, was not significantly different at every time point between WT and Δ*agr* bacteria. *n*= 10 mice/group. *N*= 2 replicates. *p-values* by two-way ANOVA, RM, with Tukey's post-test. Error bars throughout figure, mean +/- s.e.m.

a

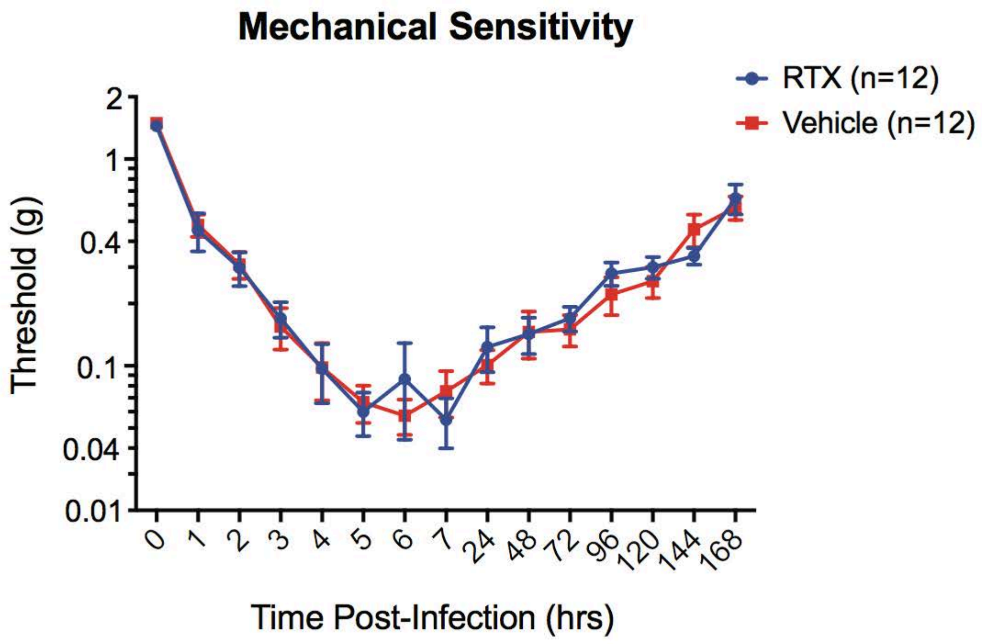

b

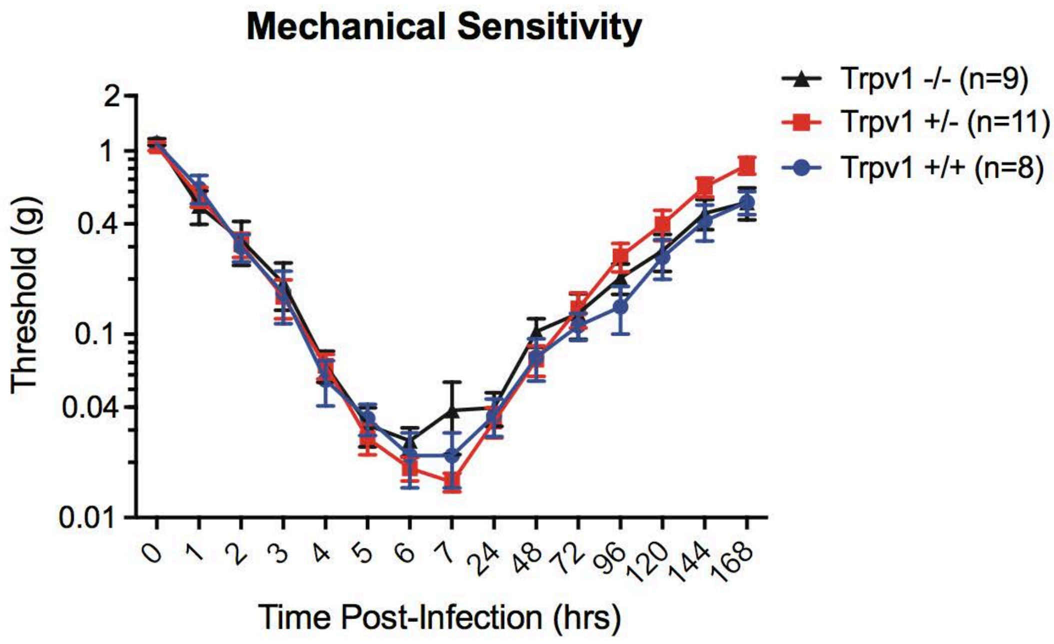

Supplemental Figure 11. TRPV1 does not mediate mechanical hyperalgesia during *S. aureus* infection. a. Mechanical hyperalgesia measured by von Frey hairs in RTX vs. vehicle treated mice following *S. aureus* infection ($1x10^6$ CFU, USA300). There is no difference in the induction of mechanical hyperalgesia between the groups as determined by two-way ANOVA with Sidak's post-tests. *n*=12 mice/group. *N*= 2 replicates. **b.** Mechanical hyperalgesia during *S. aureus* infection in $Trpv1^{-/-}$ mice compared to infected $Trpv1^{+/-}$ or $Trpv1^{+/+}$ littermates. There are no significant differences in the induction of mechanical hyperalgesia between the groups. *n*=8-11 mice/group. *N*= 3 replicates. *p*-values by two-way ANOVA with Tukey's post-test. ****, $p<0.0001$; ***, $p<0.001$; **, $p<0.01$; *, $p<0.05$. Error bars throughout figure, mean ± s.e.m.

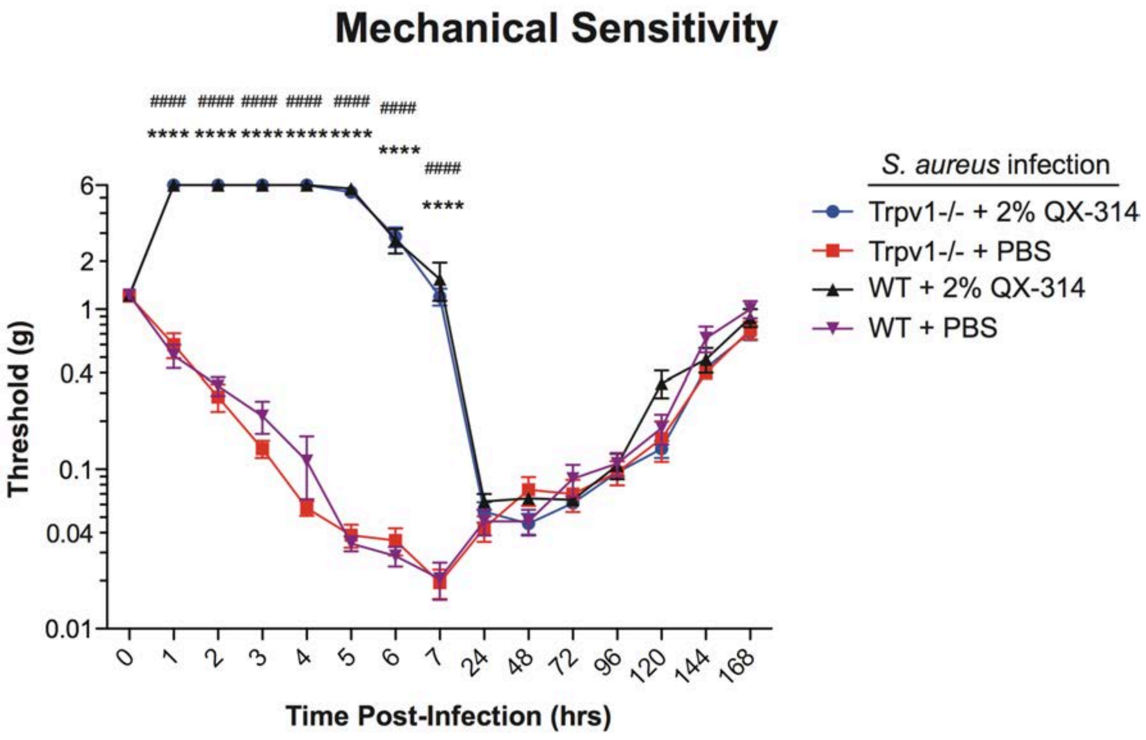

Supplemental Figure 12. TRPV1 channels are not necessary for QX-314 to block mechanical sensitivity during *S. aureus* infection. Mechanical sensitivity was measured by von Frey hairs in WT or Trpv1$^{-/-}$ mice infected with *S. aureus* (USA300, 1x10^{6} CFU) and treated with either 2% QX-314 or PBS. QX-314 significantly decreases mechanical sensitivity in both WT mice and Trpv1$^{-/-}$ mice compared to PBS treated mice of the same genotype. *Trpv1$^{-/-}$ mice: PBS treatment vs. 2% QX-314 treatment; #WT mice: PBS treatment vs. 2% QX-314 treatment. *n*=7 mice (3 females and 4 males)/group. *N*=1 replicate. *p*-values by two-way ANOVA, RM, with Tukey's post-test. **** 0.0001<*p*. Error bars, mean +/- s.e.m.
