# Supplementary material for: Staphylococcus aureus produces pain through pore-forming toxins and neuronal TRPV1 that is silenced by QX-314
Source: Nat Commun. 2018 Jan 2;9:37. doi: 10.1038/s41467-017-02448-6 (PMC5750211; doi:10.1038/s41467-017-02448-6)
Supplement: Supplementary file 3 — Description of Additional Supplementary Files [file 41467_2017_2448_MOESM3_ESM.pdf]

File Name: **Supplementary Movie 1**

Description: **Mice develop spontaneous pain following infection with USA300 *Staphylococcus aureus***. The first half of the video displays normal mouse behavior at baseline, prior to infection. The second half of the video shows spontaneous pain behavior (licking, lifting, flinching) of the right hind paw that develops 20 minutes post-infection with USA300 *S. aureus.*
